# COVID-19 Prevention: Vitamin D Is Still a Valid Remedy

**DOI:** 10.3390/jcm11226818

**Published:** 2022-11-18

**Authors:** Rachel Nicoll, Michael Y. Henein

**Affiliations:** Department of Public Health and Clinical Medicine and Heart Centre, Umea University, 901 87 Umea, Sweden

Seven meta-analyses and systematic reviews and three later clinical trials argued that low vitamin D status increased susceptibility to COVID-19 and the risk of greater disease severity and mortality [1,2,3,4,5,6,7,8,9,10]. Furthermore, there are five meta-analyses and systematic reviews of vitamin D supplementation for the prevention of acute respiratory infection (ARI) [11,12] and COVID-19 [13,14,15], as well as a later clinical trial [16], all showing that supplementation can protect against COVID-19 infection, disease severity, and death. The evidence could not be much more conclusive than this.

Consequently, it was surprising to learn about Joliffe et al.’s recent randomized controlled trial of vitamin D to prevent ARIs and COVID-19, which concluded that ‘Among people aged 16 years and older with suboptimal vitamin D status, implementation of a population level test-and-treat approach to vitamin D supplementation was not associated with a reduction in risk of all cause acute respiratory tract infection or COVID-19’ [17].

Joliffe et al.’s UK study was a test-and-treat approach used to determine the effect of correcting suboptimal vitamin D status (25-hydroxyvitamin D (25(OH)D) < 75 nmol/L) on the risk of contracting ARIs and COVID-19. Those with 25(OH)D < 75 nmol/L (30 ng/mL) were randomized to six months of supplementary vitamin D at 3200 IU/day, 800 IU/day, or no supplements. The outcome was the percentage of subjects with confirmed ARI/COVID-19.

What was different about this trial that might have caused it to fail? Analysis of Joliffe et al’s paper gives rise to a number of observations. 

Of particular importance was the treatment of participants randomized to ‘No supplementation’. Instead of being given a placebo, as would be normal in a controlled study, they were given nothing and were informed that it was a vitamin D trial, thereby alerting them to the fact that vitamin D supplementation could be an important infection preventive in the middle of the COVID-19 pandemic. As a result, almost 50% reported taking their own vitamin D supplements. We do not know what level of supplementation these participants took and we can assume that if 50% reported supplementation, the actual number was probably higher. As Dr David Grimes noted in a BMJ Rapid Response, this was therefore ‘a randomised UNCONTROLLED study’ [18]; consequently, any comparison of the intervention arm with the ‘no supplementation’ arm was rendered meaningless. The authors sought to overcome this limitation by conducting sensitivity analysis, but this is no substitute for conducting a properly controlled trial.

Furthermore, the authors took the unusual step of retesting those who had baseline vitamin D levels of ≥75 nmol/L (≥30 ng/mL) after 2 months. If they now proved to have vitamin D levels of <75 nmol/L (<30 ng/mL), they were included in the study and supplemented for four months. These new participants amounted to 11% in the lower dose group and 20% in the higher dose group, which again risks distorting the results as they would have been less likely to benefit from vitamin D, as their second attempt at a baseline level would almost certainly have been only slightly below 75 nmol/L (30 ng/mL).

Following on from the first observation, most of the results depended upon all three groups actually telling the truth about the amount of supplemented vitamin D, whereas it is well known that participants respond to questionnaires in a manner designed to minimize criticism to themselves. For example, in the intervention arm, 90.9% reported that they took supplements at least six times a week. Based on the findings of other studies, this degree of adherence seems high. According to the authors, the fact that those retested showed a significantly higher vitamin D level compared with the ‘control group’ provides ‘objective evidence of a high level of adherence’. Though it indicates some adherence, it is not possible to make this kind of judgement merely from an increase from baseline levels. Elsewhere in sensitivity analysis, it appears that 94% claimed to have taken supplements ‘more than half the time’. How much more? If they only took the supplements for half the time, this would render a dose of 3200 IU/day an effective dose of 1600 IU/day.

The authors report that not even 60% were tested for vitamin D levels at the end of the trial, but there was no sub-group analysis to determine whether the supplements raised vitamin D levels to a level shown previously to be protective against ARIs and COVID-19. Interestingly, the ‘control’ group had a mean level of 66.6 nmol/L (26.6 ng/mL), suggesting that their supplementation was probably considerable; a recent large European study found that the UK had the second lowest mean vitamin D levels at 47 nmol/L (18.8 ng/mL). Given that the mean age of the participants in this Jolliffe et al. study was >60, this mean level of 66.6 nmol/L (26.6 ng/mL) was all the more remarkable since the elderly are known to have lower vitamin D levels.

What target blood level should have been attempted in this supplementation trial? While it is clear from a meta-analysis that baseline vitamin D levels of <75 nmol/L (<30 ng/mL) were associated with increased COVID-19 infection, hospitalization, ICU admission, and mortality [19], few studies actually assess a minimum effective blood level to avoid these outcomes. Seal et al. show that the risk of hospitalization and/or mortality continues to decrease up to at least a blood level of 150 nmol/L (60 ng/mL) [20]. This was considerably higher than the level achieved in Joliffe et al’s higher dose supplementation group (102.9 nmol/L or 41.16 ng/mL). Another study by Borsche et al. conducted regression analysis to determine that zero COVID-19 mortality could be achieved at a vitamin D blood level of 125 nmol/L (50 ng/mL), again considerably higher than levels achieved in Joliffe et al’s study. The Borsche et al authors recommend raising serum vitamin D to 125 nmol/L (50 ng/mL) in order to save the most lives, even in patients with comorbidities [7].

The dosage may also have contributed to the apparent failure of this trial. Even the higher group dosage of 3200 IU/day (supposing that all participants took it every day) was considerably lower than the dosage used in many successful trials. Bergman et al. showed that 4000 IU/day given for one year was effective in preventing respiratory tract infections in those who suffered frequently [21], while 4000 IU/day for one month also achieved a lower COVID-19 infection rate, the risk reducing with increasing vitamin D levels [22], and a dose of 5000 IU/day versus 1000 IU/day in mild–moderate COVID-19 patients for two weeks reduced the recovery time for cough and gustatory sensory loss [23]. Supplementation to achieve a vitamin D blood level of 75 nmol/L (30 ng/mL) also decreased the risk of COVID-19 infection, severe disease, and mortality [24,25]. These trials suggest that either a dose of at least 4000 IU/day would be appropriate or that participants supplement to achieve a blood level of at least 125 nmol/L (50 ng/mL), as per the Borsche et al. study [7], but preferably 150 nmol/L (60 ng/mL), as per the study by Seal et al. [20]. As previously mentioned, without testing all participants at the end of the study, it is impossible to determine the true adherence to the allocated doses. Because many of these trial subjects were elderly, it is worth bearing in mind that they will need a higher dose of vitamin D for it to be effective.

An analysis of outcomes based on baseline vitamin D levels is sadly lacking. In fact, the authors state that outright vitamin D deficiency (<25 nmol/L or 10 ng/mL) at baseline was rare, and the study therefore lacked power to detect an intervention effect in this group, who are more likely to derive clinical benefit from supplementation.

In fact, Grant et al. [26] warn of the problems of designing clinical trials of vitamin D in a similar manner to randomized controlled trials (RCTs) of therapeutic drugs, through failure to recognize that vitamin D is a nutrient with a unique metabolism requiring specific consideration in trial design. They show that RCTs of vitamin D can fail for several reasons, all of which are relevant in Joliffe et al.’s study: few participants have low baseline 25(OH)D concentrations; relatively small vitamin D doses; participants ingesting other sources of vitamin D; results being analysed without consideration of 25(OH)D concentrations achieved. Grant et al recommend designing an RCT using adjustable vitamin D supplementation based on serum 25(OH)D concentrations to achieve target 25(OH)D levels, as was successfully carried out by Gönen et al. [24].

Finally, a point about vaccination. Unfortunately, the Joliffe et al. study was conducted during the vaccine roll-out. Those who had received one or more doses of the vaccine at baseline were 2.5%, while >89% had received one or more doses by the end of the study. There is no discussion of what the impact of this might have been on the results and the authors state that they did not carry out a sensitivity analysis. Nevertheless, they claim that sub-group analysis showed that there was ‘no effect of vitamin D on risk of COVID-19 either before or after COVID-19 vaccination’. Nevertheless, increasing evidence shows that vaccination inhibits both a normal innate and adaptive immune response [27,28], impairs type 1 interferon signaling [29] and increases inflammation [30,31], making individuals more susceptible to COVID-19. We can see the impact of this in two UK studies, one showing that participants with two doses of the vaccine were 44% more likely to be infected with COVID-19 more than 14 days after vaccination [32] and the other showing that vaccine effectiveness against COVID-19 turned negative after 80 days [33]. Elsewhere, a preprint paper showed that vaccination could increase risk of Omicron infection by up to 27% after five months, with negative effectiveness for three doses against four out of five Omicron subvariants, and showing that a greater number of vaccinations could give rise to a higher risk of infection [34]. A Lancet preprint study also found negative vaccine effectiveness against Omicron infection with two doses after 15 weeks and negative vaccine effectiveness against hospitalization and death after a year [35]. Furthermore, in the elderly, another preprint study found that impaired vaccine responses contributed to their increased susceptibility to COVID-19 infection [36]. These findings suggest that Jolliffe et al. were unwise to ignore vaccination as a confounding factor, since the higher risk of COVID-19 infection in the vaccinated may have rendered their relatively small vitamin D dose ineffective.

Overall, this study by Joliffe et al., represents a wasted opportunity and proposes conclusions which are not warranted by the study methodology. We consider that raising vitamin D status in those with sub-optimal levels remains a valid means of protection against ARIs and COVID-19.
